# Triglyceride glucose index influences platelet reactivity in acute ischemic stroke patients

**DOI:** 10.1186/s12883-021-02443-x

**Published:** 2021-10-26

**Authors:** Yinping Guo, Jing Zhao, Yi Zhang, Lingshan Wu, Zhiyuan Yu, Dan He, Hao Huang, Wensheng Qu, Xiang Luo

**Affiliations:** 1 Department of Neurology, Tongji Hospital, Tongji Medical College, Huazhong University of Science and Technology, Wuhan, Hubei 430030 People’s Republic of China; 2 Department of Neurology, The First Affiliated Hospital, Sun Yat-sen University, Guangzhou, Guangdong China; 3 Guangdong Provincial Key Laboratory for Diagnosis and Treatment of Major Neurological Diseases, National Key Clinical Department and Key Discipline of Neurology, Guangzhou, Guangdong China

**Keywords:** Acute ischemic stroke, Dual antiplatelet therapy, Insulin resistance, TyG index

## Abstract

**Aim:**

Insulin resistance was reported to increase the risk of ischemic stroke, which can be assessed by the triglyceride glucose (TyG) index. However, it remains unclear whether the TyG index influences the platelet reactivity during the treatment of ischemic patients.

**Methods:**

Ischemic stroke patients receiving dual antiplatelet therapy (DAPT) within 48 h onset were consecutively included. The TyG index was calculated as ln (fasting triglyceride [mg/dL] × fasting glucose [mg/dL]/2). The top quartile of TyG index was defined as insulin resistance. The platelet reactivity was assessed by thromboelastography. The platelet inhibition rate induced by arachidonic acid (AA) or adenosine diphosphate (ADP) was used to confirm the high residual on-treatment platelet reactivity (HRPR) to aspirin or clopidogrel, respectively. The association between TyG index and platelet reactivity was assessed by Kruskal–Wallis test. The independent risk factors of HRPR were determined by multivariate logistic regression analysis.

**Results:**

A total of 1002 patients were included and divided into 4 groups by quartiles of the TyG index (< 2.02; 2.02–2.27; 2.27–2.52; ≥2.52). The findings demonstrated that the maximum intensity of the clot increased, but the AA-induced platelet inhibition rate decreased, depending on the TyG index quartiles. No significant difference was found in the ADP-induced platelet inhibition rate among groups. The prevalence of aspirin HRPR increased depending on the TyG index quartile. Unlike the non-insulin resistance group, the insulin resistance group was independently associated with aspirin HRPR (OR = 1.689, 95% CI 1.14 to 2.51, *P* = 0.009).

**Conclusions:**

In acute ischemic stroke patients taking DAPT, the elevation of the TyG index is associated with enhanced platelet reactivity and higher prevalence of aspirin HRPR. Insulin resistance assessed by the TyG index could be an independent risk factor for aspirin HRPR.

## Background

Ischemic stroke is an important health problem worldwide and has the characteristics of high mortality and a high disability rate. Reducing the incidence of stroke and improving the prognosis of stroke are problems that urgently need to be solved. Aspirin and clopidogrel are widely used in the acute phase treatment and secondary prevention of ischemic stroke [1, 2]. They not only significantly reduce the mortality and disability rate but also effectively prevent the recurrence of stroke [3]. However, some patients with ischemic stroke will experience new ischemic events despite having received antiplatelet therapy [4–6]. One possible reason is the high residual on-treatment platelet reactivity (HRPR), which means a reduced platelet inhibition rate and the absence of an antiplatelet effect [7].

Insulin resistance is considered to be a significant risk factor of metabolic disorders [8], diabetes mellitus [9, 10], and atherosclerotic disease [11, 12]. In addition, more and more evidence shows that insulin resistance is common in ischemic stroke patients [13, 14]. Insulin resistance is independently related to the adverse clinical consequences of ischemic stroke, which can exacerbate the neurologic worsening during hospitalization and trigger the recurrence of ischemic stroke [15, 16]. At present, the correlation between insulin resistance and platelet reactivity in acute ischemic stroke patients treated with dual antiplatelet therapy (DAPT) is unknown.

The gold-standard method for diagnosing insulin resistance is the hyperinsulinemic-euglycemic clamp (HIEC), which is not commonly used due to its cost and complexity [17]. Although widely used in research, the homeostatic model for assessing insulin resistance (HOMA-IR) is rarely used in clinical practice because of the nonuniform standards. Relatively, the triglyceride glucose index (TyG index) is a simple, economical, and reliable evaluation index of insulin resistance [18, 19]. It has been confirmed that the TyG index is significantly coincident with HIEC and HOMA-IR in both non-diabetic and diabetic patients [20, 21]. Assessed by the TyG index, insulin resistance was reported to be associated with carotid atherosclerosis, [22] coronary artery calcification [23], and ischemic stroke [13]. However, the correlation between the TyG index and the platelet reactivity in acute ischemic stroke patients receiving DAPT is unclear.

In order to disclose the impact of insulin resistance on HRPR, the correlation between the TyG index and platelet reactivity was evaluated in acute ischemic stroke patients who were receiving DAPT. The results may provide evidence for more effective individualized treatments during antiplatelet therapy.

## Methods

### Study population

Ischemic stroke patients who were admitted into the neurological department of Tongji Hospital were included retrospectively, from September 2013 and May 2019. Patients were included if: (1) over 18 years old; (2) with a diagnosis of acute ischemic stroke according to clinical symptoms and imaging (magnetic resonance/computer tomography); (3) receiving DAPT (clopidogrel 75 mg/day and aspirin 100 mg /day without loading dose) within 48 h of symptom onset, according to guidelines when diagnosed as acute minor ischemic stroke (with a National Institutes of Health Stroke Scale (NIHSS) score ≤ 3) [24], high-risk transient ischemic attack (TIA, with a ABCD2 score ≥ 4), or symptomatic severe stenosis (70–99%) of a major intracranial artery (middle cerebral, carotid, vertebral, or basilar arteries) [2, 25], without evidences of cardioembolism. The exclusion criteria were as follows: (1) Without platelet reactivity testing after DAPT; (2) without fasting plasma glucose (FPG) or fasting triglycerides before DAPT; (3) any medications taken within the past 3 months that may affect blood coagulation function, such as cilostazol, warfarin, dabigatran, heparin, or factor Xa inhibitors (such as rivaroxaban); and (4) a history of malignant tumors, digestive diseases, or severe liver/ kidney/ blood-related diseases.

This study has been approved by the Tongji Hospital Ethics Committee (No. TJ-IRB20210107) and conducted according to the Declaration of Helsinki. According to ethic guidelines, the requirement of informed consent was waived since this study had no potential to harm the rights or welfare of subjects.

### Clinical assessments

Clinical data included age, sex, smoking (defined as ≥1 cigarette per day for 1 year or more), alcohol intake (defined as weekly alcohol intake exceeding 200 g for 1 year or more), a history of ischemic stroke/TIA, hypertension, hyperlipidemia, coronary heart disease (referring to prior angina pectoris or myocardial infarction), and diabetes mellitus (referring to glycosylated hemoglobin A1c (HbA1c) ≥ 6.5%, or two-hour plasma glucose ≥11.1 mmol/L in an oral glucose tolerance test, or self-reported history of diabetes mellitus) [2, 26, 27]. Laboratory data included serum levels of fasting triglycerides (TGs), total cholesterol, high-density lipoprotein cholesterol (HDL-C), low-density lipoprotein cholesterol (LDL-C), creatinine, glomerular filtration rate (eGFR), platelet indexes, fasting plasma glucose (FPG), and HbA1c. All of these data were tested in a standard manner in the laboratory of the hospital within 24 h enrollment and before the DAPT.

The TyG index is calculated by the formula TyG index = LN [fasting TG (mg/dL) × FPG (mg/dL)/2] [20]. The top quartile (Q4) of the TyG index is defined as insulin resistance.

### Platelet reactivity assessment

It is believed that the inhibition plateau of platelet aggregation appeared 7 days after a regular dose of either aspirin or clopidogrel. Therefore, peripheral venous whole blood was collected 7 days later after the initiation DAPT with vacutainer tubes containing 3.2% sodium citrate and sodium heparin (Becton–Dickinson, San Jose, CA). The platelet reactivity was evaluated using Thromboelastography (TEG) Analyzer 5000 (Haemonetics Corporation, USA) within 1 h after the sample collection. The platelet reactivity induced by arachidonic acid (AA) and adenosine diphosphate (ADP) activators was tested according to the manufacturer’s instructions. The maximum amplitude (MA) is the maximum intensity of the clot, which represents the maximum platelet function that can be stimulated in the blood sample. The MA_ADP_ represents the ADP-induced clot strength while the MA_AA_ represents that was induced by AA. The MA_fibrin_ represents the activator-induced clot strength (measurement of fibrin contribution) while the MA_thrombin_ represents that was induced by thrombin. The platelet inhibition rate induced by AA or ADP was calculated using the following formula: inhibition rate (%) = [(MA_thrombin_ – MA_ADP_ or MA_AA_)/(MA_thrombin_ – MA_fibrin_)] × 100%. Aspirin HRPR was defined as AA% < 50, and clopidogrel HRPR was defined as ADP% < 30 or MA_ADP_ > 47 mm [28, 29].

### Statistical analysis

Statistical analysis was completed using IBM SPSS 22.0 software (IBM Corp., Armonk, NY, USA). *P* < 0.05 was considered as statistically significant difference. Categorical variables are shown as frequencies (percentages) and continuous variables as medians [interquartile range] for data with a skewed distribution. Patients were grouped by the TyG index quartiles and the clinical data were compared among groups using the Chi-square test or Kruskal–Wallis test.

For further analysis, patients were divided into aspirin HRPR and non-HRPR groups. Subsequently, the Chi-square or Mann–Whitney *U* test was used to compare the demographic and clinical characteristics between groups. Potential confounders were adjusted by multivariable logistic models. The results were shown as adjusted odds ratios (ORs) with 95% confidence intervals (CIs).

## Results

### Demographics and clinical characteristics

**Fig. 1:**
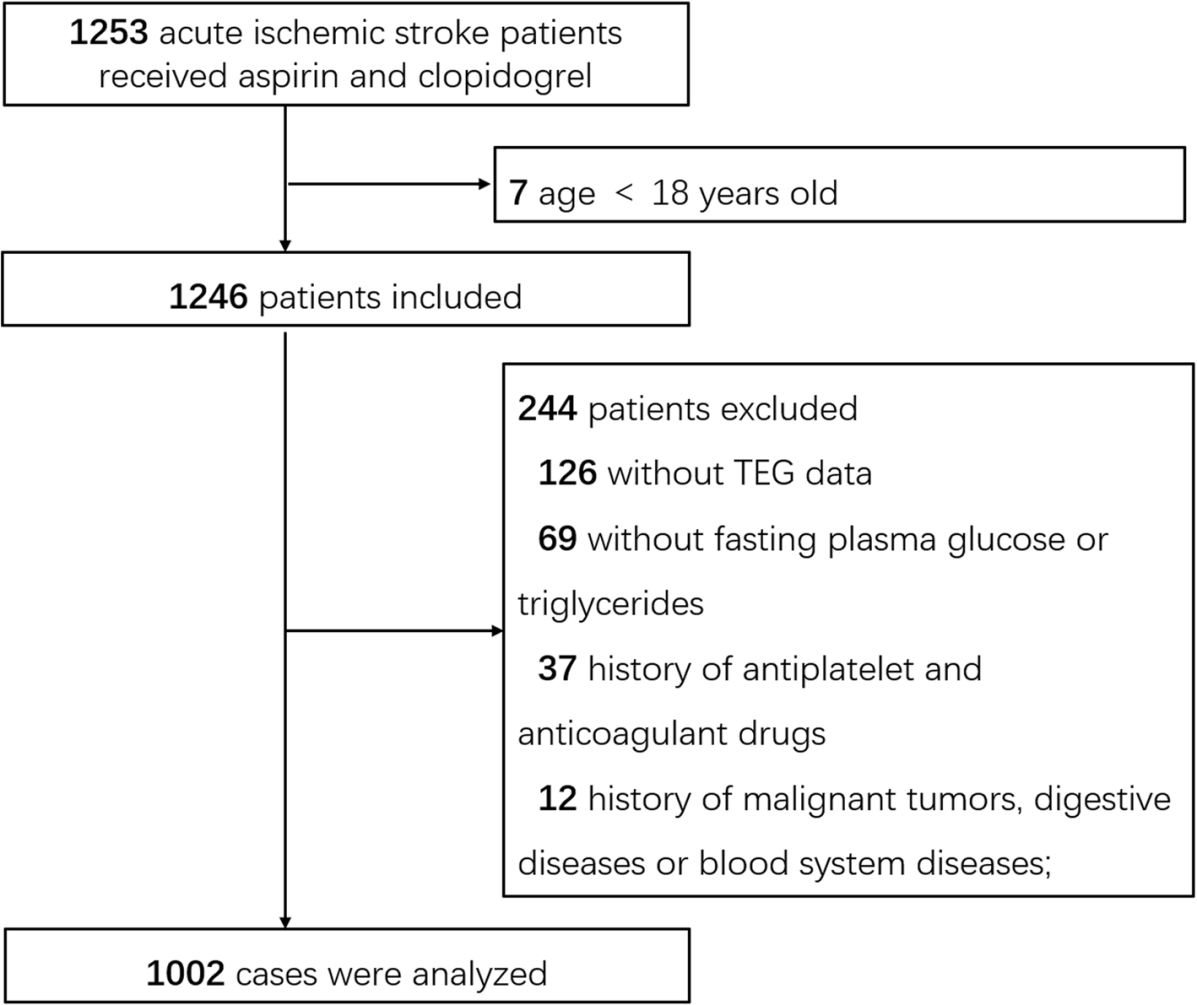
Flowchart of patient selection. TEG: Thromboelastography

In the present study, 1002 patients were analyzed (Fig. 1). Values of the TyG index were divided into quartiles: Q1: TyG < 2.02 (*n* = 250); Q2: TyG = 2.02–2.27 (*n* = 251); Q3: TyG = 2.27–2.52 (*n* = 251); and Q4: TyG ≥ 2.52 (*n* = 250).

The baseline demographics and clinical characteristics of patients are described in Table 1. Comparing among different quartiles, a higher TyG index was related to a higher percentage of males, a history of diabetes mellitus, non-smoking cohorts, as well as the elevation of triglycerides, total cholesterol, HDL-C, LDL-C, creatinine, platelet count, fasting glucose, and HbA1c (*P* < 0.05).

**Table 1:** Patient baseline characteristics according to TyG index quartiles

Characteristics	Q1 (< 2.02) (***n*** = 250)	Q2 (2.02–2.27) (***n*** = 251)	Q3 (2.27–2.52) (***n*** = 251)	Q4 (≥2.52) (***n*** = 250)	***P*** Value
Age, y	57.0 [50.0–65.0]	57.0 [50.0–65.0]	58.0 [50.0–65.0]	58.0 [52.0–64.3]	0.737
Male, *n* (%)	207 (82.8)	183 (72.9)	170 (67.7)	166 (66.4)	**< 0.001**
Smoking, *n* (%)	140 (56.0)	126 (50.2)	112 (44.6)	113 (45.2)	**0.038**
Alcohol intake, *n* (%)	117 (46.8)	106 (42.2)	95 (37.8)	95 (38.0)	0.135
**Medical history,** ***n*** **(%)**					
History of stroke/TIA	49 (19.6)	47 (18.7)	39 (15.5)	55 (22.0)	0.322
Hypertension	173 (69.2)	158 (62.9)	177 (70.5)	170 (68.0)	0.288
Diabetes mellitus	64 (25.6)	55 (21.9)	70 (27.9)	149 (59.6)	**< 0.001**
Hyperlipidemia	21 (8.4)	27 (10.8)	27 (10.8)	37 (14.8)	0.149
Coronary heart disease	23 (9.2)	16 (6.4)	22 (8.8)	26 (10.4)	0.440
**Biochemical indexes**					
Triglyceride (mmol/L)	2.5 [2.2–2.9]	3.3 [3.1–3.7]	4.0 [3.5–4.3]	4.6 [3.9–5.2]	**< 0.001**
Total cholesterol (mmol/L)	1.0 [0.8–1.3]	1.2 [0.9–1.7]	1.4 [1.1–1.9]	1.6 [1.1–2.3]	**< 0.001**
HDL-C (mmol/L)	0.9 [0.7–1.0]	0.9 [0.8–1.1]	0.9 [0.8–1.1]	1.0 [0.8–1.1]	**< 0.001**
LDL-C (mmol/L)	1.3 [1.1–1.6]	2.0 [1.7–2.3]	2.5 [2.1–2.9]	2.9 [2.3–3.6]	**< 0.001**
Creatinine (μmol/L)	74.0 [65.0–87.0]	71.0 [61.0–83.0]	72.0 [62.0–83.0]	71.0 [59.0–84.0]	**0.041**
eGFR (mL/min/1.73m^2^)	94.8 [80.6–105]	96.1 [84.9–104]	92.3 [83.1–103]	95.4 [81.4–103]	0.580
Platelet count (× 10^9^/L)	211 [169–247]	209 [175–258]	211 [178–250]	220 [184–259]	**0.039**
PDW (fL)	13.5 [11.9–15.1]	13.1 [11.7–14.9]	13.3 [11.9–15.3]	13.2 [11.7–14.9]	0.617
P-LCR (%)	33.8 [28.2–40.4]	32.4 [27.0–39.7]	32.0 [26.6–39.7]	32.9 [27.0–38.5]	0.210
Fasting glucose (mmol/L)	4.9 [4.6–5.3]	5.1 [4.8–5.5]	5.4 [5.1–6.1]	7.1 [5.8–10.0]	**< 0.001**
HbA1c (%)	5.7 [5.5–6.2]	5.7 [5.5–6.1]	5.8 [5.5–6.3]	6.7 [5.8–10.3]	**< 0.001**

Data given as *n* (%) or median [interquartile range]

Abbreviations: *TyG index* triglyceride glucose index, *Q1–Q4* TyG index quartiles, *TIA* transient ischemic attack, *HDL-C* high-density lipoprotein cholesterol, *LDL-C* low-density lipoprotein cholesterol, *eGFR* glomerular filtration rate, *PDW* platelet distribution width, *P-LCR* platelet large cell ratio, *HbAc1* glycosylated hemoglobin A1c

### Relationship between TyG index and platelet reactivity

Comparisons among different quartiles revealed that patients with a higher TyG index had higher levels of MA (the median values were 61.8, 62.1, 62.7, and 63.1 mm in each quartile respectively, *P* = 0.004) and lower levels of AA% (the median values were 99.0, 97.4, 96.2, and 93.0% in each quartile respectively, *P* < 0.001). However, there was no significant difference of either MA_ADP_ or ADP% levels among the quartiles (Table 2).

**Table 2:** Platelet reactivity and HRPR according to TyG index quartiles

Characteristics	Q1 (< 2.02) (***n*** = 250)	Q2 (2.02–2.27) (***n*** = 251)	Q3 (2.27–2.52) (***n*** = 251)	Q4 (≥2.52) (***n*** = 250)	***P*** Value
MA (mm)	61.8 [57.8–65.2]	62.1 [58.2–65.5]	62.7 [59.2–66.3]	63.1 [59.7–66.5]	**0.004**
MA_ADP_ (mm)	30.9 [18.1–42.5]	31.0 [16.8–41.3]	31.6 [18.6–42.5]	30.0 [18.3–42.2]	0.645
ADP% (%)	58.4 [39.1–81.4]	60.2 [40.1–85.1]	58.2 [35.5–82.3]	61.6 [41.9–86.0]	0.306
AA% (%)	99.0 [87.2–100]	97.4 [83.7–100]	96.2 [77.9–100]	93.0 [70.0–98.9]	**< 0.001**
Clopidogrel HRPR, n (%)	40 (16.0)	43 (17.1)	54 (21.5)	51 (20.4)	0.338
Aspirin HRPR, n (%)	18 (7.2)	30 (12.0)	37 (14.7)	45 (18.0)	**0.003**

Data given as *n* (%) or median [interquartile range]

Abbreviations: *TyG index* triglyceride glucose index, *Q1–Q4* TyG index quartiles, *MA* maximum amplitude, *MA*_*ADP*_ ADP-induced platelet-fibrin clot maximum amplitude, *ADP%* inhibition rate of adenosine diphosphate (ADP), *AA%* inhibition rate of arachidonic acid (AA), *HRPR* high residual on-treatment platelet reactivity

There were 12.97% patients who were identified as having aspirin HRPR. The prevalence of aspirin HRPR was increased in the higher quartile of the TyG index (7.2% vs. 12.0% vs. 14.7% vs. 18.0%, *P* = 0.003). Relatively, there was no significant difference in the prevalence of the clopidogrel HRPR among the TyG index quartiles (Table 2).

### Risk factors of aspirin HRPR

To disclose the risk factors for aspirin HRPR, the included patients were divided into two groups, non-HRPR and HRPR. The baseline demographic data, clinical characteristics, and laboratory data were compared. It demonstrated that the history of stroke/TIA, triglycerides, LDL-C, fasting glucose, and TyG index quartiles were significantly different between groups (Table 3). After adjustment for the potential confounders (history of stroke/TIA), compared with that in the Q1 quartile, the TyG index in either Q2, Q3 or Q4 was independently related to the aspirin HRPR in acute ischemic stroke patients taking DAPT (Q2: OR = 1.76, 95% CI 0.95–3.26; Q3: OR = 2.29, 95% CI 1.27–4.16; Q4: OR = 2.81, 95% CI 1.57–5.01; *P* < 0.05). Moreover, insulin resistance, presented by the Q4 quartile of the TyG index, was confirmed as an independent risk factor for the aspirin HRPR when comparing with the non-insulin resistance cohort (Q1+ Q2 + Q3) (OR = 1.69, 95% CI 1.14–2.51, *P* = 0.009).

**Table 3:** Risk factors for aspirin HRPR

Characteristics	Non-HRPR (***n*** = 872)	HRPR (***n*** = 130)	***P*** Value
Age (y)	57.0 [50.0–65.0]	58.0 [50.8–66.0]	0.545
Male, *n* (%)	632 (72.5)	94 (72.3)	0.968
Smoking, *n* (%)	428 (49.1)	63 (48.5)	0.895
Alcohol intake, *n* (%)	361 (41.4)	52 (40.0)	0.763
**Medical history,** ***n*** **(%)**			
History of stroke/TIA	155 (17.8)	35 (26.9)	**0.013**
Hypertension	591 (67.8)	87 (66.9)	0.846
Diabetes mellitus	295 (33.8)	43 (33.1)	0.865
Hyperlipidemia	99 (11.4)	13 (10.0)	0.648
Coronary heart disease	77 (8.80)	10 (7.70)	0.667
**Laboratory data**			
Triglyceride (mmol/L)	3.46 [2.81–4.18]	3.84 [3.09–4.40]	**0.001**
Total cholesterol (mmol/L)	1.23 [0.92–1.07]	1.30 [1.02–1.88]	0.141
HDL-C (mmol/L)	0.92 [0.79–1.07]	0.92 [0.77–1.09]	0.902
LDL-C (mmol/L)	2.08 [1.49–2.68]	2.33 [1.80–3.00]	**0.001**
Creatinine (μmol/L)	72.0 [62.0–85.0]	73.0 [62.8–83.0]	0.763
eGFR (mL/min/1.73m^2^)	94.4 [82.5–103]	94.4[80.9–105]	0.891
Platelet count (×10^9^/L)	211 [177–251]	221 [180–264]	0.060
PDW (fL)	13.3 [11.8–15.0]	13.3 [11.9–15.1]	0.935
P-LCR (%)	33.0 [27.3–39.7]	32.1 [27.0–38.3]	0.593
Fasting glucose (mmol/L)	5.29 [4.84–6.29]	5.55 [5.05–6.75]	**0.010**
HbA1c (%)	5.80 [5.50–6.50]	5.90 [5.60–6.80]	0.297
MA (mm)	62.4 [58.7–66.0]	63.6 [58.2–66.1]	0.337
TyG index quartiles			**< 0.001**
Q1 (< 2.02)	232 (26.6)	18 (13.8)	
Q2 (2.02–2.27)	221 (25.3)	30 (23.1)	
Q3 (2.27–2.52)	214 (24.5)	37 (28.5)	
Q4 (≥2.52)	205 (23.5)	45 (34.6)	

Data given as *n* (%) or median [interquartile range]

Abbreviations: *TIA* transient ischemic attack, *HDL-C* high-density lipoprotein cholesterol, *LDL-C* low-density lipoprotein cholesterol, *eGFR* glomerular filtration rate, *PDW* platelet distribution width, *P-LCR* platelet large cell ratio, *HbAc1* glycosylated hemoglobin A1c, *MA* maximum amplitude, *TyG index* triglyceride glucose index, *Q1–Q4* TyG index quartiles

## Discussion

In the present study, it was found that as the TyG index increased, the prevalence of diabetes, blood lipids, and glucose levels were elevated significantly. The TyG index was significantly correlated with the increased platelet reactivity and decreased response to aspirin in acute ischemic stroke patients who received DAPT; the insulin resistance was an independent risk factor for aspirin HRPR. This suggested that insulin resistance may increase the progression and recurrence of ischemic stroke by decreasing the platelet reactivity to aspirin in patients receiving DAPT.

It is well known that insulin resistance is closely related to hyperglycemia and abnormal lipid metabolism. In the process of body glucose balance, insulin resistance affects the expression and activity of glucose transporters, thereby increasing the accumulation of glucose in the circulatory system and forming hyperglycemia [30]. Similarly, during the process of lipid metabolism, insulin resistance increases the synthesis of triglycerides and their release, leading to increased triglycerides in plasma [31]. Both high blood glucose and abnormal lipid metabolism can affect insulin activity and exacerbate the insulin resistance. The TyG index has been proved to consistent with the “gold standard” for diagnosing insulin resistance, HIEC [17]. In the present study, it was demonstrated that a higher TyG index is related to diabetes mellitus, triglycerides, total cholesterol, and LDL-C, which suggests that the TyG index is a reliable alternative index for evaluating the insulin resistance.

Previous studies have found that insulin resistance is usually associated with platelet activation [32–34]. Insulin resistance could cause not only chronic inflammation but also endothelial dysfunction, which contributes to the increase of platelet adhesion and aggregation. In addition, studies have found that, by improving insulin resistance, the use of insulin sensitizers or strict blood sugar control could reduce the risk of thrombosis [35, 36]. In the present study, the highest levels of MA, representing increased platelet activation, were found in the highest quartile of the TyG index (insulin resistance group). However, further research is still needed to clarify the mechanism.

Previous studies have found that aspirin can decrease the risk of ischemic events by almost 10% or 20% in patients with or without diabetes, respectively [37]. Relative to non-diabetic patients, aspirin is less effective at the prevention of cerebrovascular diseases in diabetic patients [38]. The aspirin HRPR is thought to contribute to this variation. Thromboxane A2 is an important positive feedback mediator involved in platelet activation. However, aspirin could irreversibly inhibit the transformation of arachidonic acid to thromboxane A2, leading to the reduction of platelet aggregation [39]. Previous studies have shown that insulin resistance could activate other regulatory pathways of platelets, instead of thromboxane A2, to reverse the platelet inhibition of aspirin [40, 41]. Here, it was found that patients with a higher TyG index had higher levels of AA%. Insulin resistance, presented by the highest quartile of the TyG index, was an independent risk factor for aspirin HRPR, which was supported by a previous study using a different method [42].

It is still controversial whether insulin resistance can influence the clopidogrel HRPR. In one previous study involving 237 patients with recent ischemic stroke or TIA receiving aspirin or/and clopidogrel, insulin resistance assessed by HOMA-IR was found to be related to HRPR, which was not specially restricted by clopidogrel resistance [43]. In another study including 66 ischemic stroke or TIA patients with clopidogrel treatment, insulin resistance assessed by HOMA-IR was associated with clopidogrel resistance tested by multiple electrode aggregometry [44]. The present study demonstrated that insulin resistance cannot influence the clopidogrel HRPR significantly. Relative to aspirin, clopidogrel resistance is more easily affected by confounding factors such as genes polymorphisms, which made it difficult to correlate insulin resistance with clopidogrel resistance. Further study of their relationship is needed.

Some limitations of this study should be addressed. First, since it was a retrospective study, some potential confounding factors for platelet reactivity, such as proton pump inhibitors, statins, or heterogeneity of metabolic genes, could not been evaluated. Second, measurement error of the system could not be completely ruled out since only one fasting test of triglyceride and glucose was performed. Third, the research subjects were from a cohort of Chinese patients, so extending these results to other cohorts may require careful interpretation and further researches.

## Conclusion

In conclusion, we found that in acute ischemic stroke patients taking DAPT, the TyG index is associated with increased platelet activity and elevated prevalence of aspirin HRPR. The insulin resistance assessed by the TyG index could be an independent risk factor for aspirin HRPR. These results partly explained the higher risk of stroke recurrence in patients with insulin resistance despite receiving intensive antiplatelet therapy. Hence, personalized treatment strategies should be implemented by considering the TyG index in order to reduce the recurrence of ischemic events.

## Data Availability

All data generated or analyzed during this study are included in this published article.

## Abbreviations

DAPT: Dual Antiplatelet Therapy
TyG: Triglyceride glucose
HRPR: High High residual on-treatment platelet reactivity
ADP: Adenosine Diphosphate
AA: Arachidonic Acid
TIA: Transient Ischemic Attack
HIEC: Hyperinsulinemic-euglycemic clamp
HOMAIR: Homeostatic model for assessing insulin resistance
FPG: Fasting plasma glucose
HDL-C: High-density lipoprotein cholesterol
LDL-C: Low-density lipoprotein cholesterol
eGFR: Glomerular filtration rate
PDW: Platelet distribution width
P-LCR: Platelet large cell ratio
HbAc1: Glycosylated hemoglobin A1c
MA: Maximum amplitude
